# Identification of Electrode Respiring, Hydrocarbonoclastic Bacterial Strain *Stenotrophomonas maltophilia* MK2 Highlights the Untapped Potential for Environmental Bioremediation

**DOI:** 10.3389/fmicb.2016.01965

**Published:** 2016-12-09

**Authors:** Krishnaveni Venkidusamy, Mallavarapu Megharaj

**Affiliations:** ^1^Centre for Environmental Risk Assessment and Remediation, University of South AustraliaMawson Lakes, SA, Australia; ^2^Cooperative Research Centre for Contamination Assessment and Remediation of the EnvironmentMawson Lakes, SA, Australia; ^3^Global Centre for Environmental Remediation, The University of NewcastleCallaghan, NSW, Australia

**Keywords:** electrode respiring bacteria, microbial electrochemical remediation systems, *Stenotrophomonas maltophilia* MK2, facultative hydrocarbon degradation, dye decolorization, catabolic genes (*alk*B, *rub*A)

## Abstract

Electrode respiring bacteria (ERB) possess a great potential for many biotechnological applications such as microbial electrochemical remediation systems (MERS) because of their exoelectrogenic capabilities to degrade xenobiotic pollutants. Very few ERB have been isolated from MERS, those exhibited a bioremediation potential toward organic contaminants. Here we report once such bacterial strain, *Stenotrophomonas maltophilia* MK2, a facultative anaerobic bacterium isolated from a hydrocarbon fed MERS, showed a potent hydrocarbonoclastic behavior under aerobic and anaerobic environments. Distinct properties of the strain MK2 were anaerobic fermentation of the amino acids, electrode respiration, anaerobic nitrate reduction and the ability to metabolize n-alkane components (C8–C36) of petroleum hydrocarbons (PH) including the biomarkers, pristine and phytane. The characteristic of diazoic dye decolorization was used as a criterion for pre-screening the possible electrochemically active microbial candidates. Bioelectricity generation with concomitant dye decolorization in MERS showed that the strain is electrochemically active. In acetate fed microbial fuel cells (MFCs), maximum current density of 273 ± 8 mA/m^2^ (1000 Ω) was produced (power density 113 ± 7 mW/m^2^) by strain MK2 with a coulombic efficiency of 34.8%. Further, the presence of possible alkane hydroxylase genes (*alk*B and *rub*A) in the strain MK2 indicated that the genes involved in hydrocarbon degradation are of diverse origin. Such observations demonstrated the potential of facultative hydrocarbon degradation in contaminated environments. Identification of such a novel petrochemical hydrocarbon degrading ERB is likely to offer a new route to the sustainable bioremedial process of source zone contamination with simultaneous energy generation through MERS.

## Introduction

Due to their toxicity and ubiquitous nature, petroleum hydrocarbons (PH) are of serious concern to the environmental and public health. Of these PH contaminants, diesel range organics (DRO) is constitute one of the most prevalent organic pollutants that are biodegradable in various environments. Medium chain hydrocarbons from octane to the long chain hydrocarbon dotriacontane are the constituents of DRO. They are usually assumed to be the fractional middle distillate of crude oil and are known to be highly noxious, hazardous, and carcinogenic (Chilcott, 2011). Increasing anthropogenic activities of these compounds leading to spillages, and leakages from underground storage tanks constitute the two dominant sources of penetration of DRO compounds from surface soils to subsurface. As an ultimate result, DRO became the most encountered environmental pollutants in groundwater and soils (Gallego et al., 2001). Consequently, horizons of subsoil, aquifer and groundwater systems are prone to long-term contamination of these hydrophobic contaminants. Microbial clean-up of these DRO compounds is claimed to be an efficient, economical, and versatile alternative to the established physicochemical treatments that are prone to cause recontamination by secondary contaminants (Hong et al., 2005; Megharaj et al., 2011). The biodegradation of these compounds at the surface has been well documented for a century whereas subsurface biodegradation awaits further research on deeper insights into the metabolic activities involved and the extent and rate of hydrocarbon degradation (Röling et al., 2003). Subsurface hydrocarbon contaminated reservoirs are primarily dominated by obligate and facultative anaerobic microbial communities. These microbial communities can adjust their metabolism to take account of the availability of final electron acceptors and can have more complex enzymatic systems involved in the degradation of contaminants. However, the rate of microbial utilization of these PH compounds is very slow especially under anaerobic environments where the availability of relevant electron acceptors is limited (Morris et al., 2009).

Recent research on removal of such recalcitrant contaminants using advanced microbial electrochemical systems is gaining new interest in its practical applications involved in subsurface hydrocarbon bioremediation. These microbial electrochemical remediation systems (MERS) transform the chemical energy available in organic pollutants into electrical energy by capitalizing on the biocatalytic potential of a peculiar group of microbes called “electric communities” (Logan, 2008; Morris et al., 2009). These electric microbial communities have received much attention in the field of electromicrobiology because of their exoelectrogenic capabilities to degrade substrates that range from easily degradable natural organic compounds to xenobiotic compounds such as PH contaminants (Venkidusamy and Megharaj, 2016; Venkidusamy et al., 2016; Zhou et al., 2016). Many studies have shown the predominance of many strains and species of *Geobacter* in microbial fuel cells (MFCs) fed with different types of substrates. However, the microbial community composition is divergent in MERS (Morris et al., 2009; Venkidusamy et al., 2016), and the physiology of such populations remains to be explored in detail. The identification of such bacterial population with dual functions of electrode respiration and petrochemical degradation highlights the biotechnological potential involved in sustainable remediation of PH contaminated sites and MERS. We have therefore attempted to (i) find representative microbial candidates with such abilities of hydrocarbonoclastic electrode respiration through the anode enrichment of MERS, (ii) demonstrate the bioremediation potential of isolated bacteria to completely mineralize DRO compounds in anoxic environments and (iii) also investigate the presence of catabolic genes responsible for hydrocarbon degradation in these bacteria.

## Materials and methods

### Source of chemicals

Refined fossil fuels such as DRO and other PH products used throughout the study were obtained from local BP outlet (Australia). Aliphatic hydrocarbon standards, solvents such as hexane and methylene chloride, redox indicators such as 2–6, dichlorophenol indophenol (DCPIP), and tetrazolium violet (2, 5-diphenyl-3-[α-naphthyl] tetrazolium chloride) and diazo dyes were purchased from Sigma Aldrich Trading Co. Ltd (Australia). All the solvents used were of HPLC grade.

### Bacterial strain, media, and culture conditions

The bacterial strain MK2 was isolated from the anodic biofilm of a MERS fed with hydrocarbons operated in a fed-batch mode over a period of 12 months. Hydrocarbons contaminated groundwater (RAAF Base, Williamstown, NSW, Australia) and activated sludge (WTP, South Australia) served as inoculum for these PH fed MERS. Bacterial cells from the anodic biofilm were extracted into a sterile phosphate buffer and shaken vigorously to separate cells from the electrode. Aliquots of the extracted cell suspensions were serially diluted and plated onto mineral salt medium (MSM) agar (Grishchenkov et al., 2000) containing 1% DRO compounds and incubated for 3 weeks. Single colonies were selected and transferred to Luria Bertani (LB) agar plates. Unless otherwise stated all incubations were performed at room temperature. Media used throughout the study were Bushnell Hass (Hanson et al., 1993), mineral salts medium (Grishchenkov et al., 2000) and Luria-Bertani medium (Sambrook et al., 1989). Nitrate served as the terminal electron acceptor in anaerobic biodegradation experiments. A chemically defined medium supplemented with Wolfe's trace elements and vitamins was used in the microbial electrochemical studies as previously described (Oh et al., 2004). One liter of growth medium contains (g l^−1^) KCl 0.13, Na_2_HPO_4_ 4.09, NaH_2_PO_4_ 2.544, NH_4_Cl 0.31. The pH of the medium was adjusted to 7 ± 0.2 and further fortified with Wolfe's trace elements and vitamins. The purified strain was stored in glycerol: Bushnell Hass broth and glycerol: Luria-Bertani broth (1:20) at −80°C. Biolog-GN2 (Biolog., USA) plates were used to determine the utilization of various carbon sources according to the manufacturer's instructions.

### Bacterial 16S rRNA gene sequencing

Genomic DNA of strain MK2 was extracted from aerobically grown cells using the UltraClean microbial DNA isolation kit (MO BIO, CA, USA) following the manufacturer's instructions. The polymerase chain reaction (PCR) mediated amplification of 16S rRNA gene fragments was performed using the combination of universal primers, E8F (5′-AGAGTTTGATCCTGGCTCAG3′) and 1541R (5′AAGGAGGTGATCCANCCRCA 3′) (Weisburg et al., 1991). The PCR products were purified using the UltraClean PCR clean-up kit (Mo Bio, Carlsbad, CA, USA) following the manufacturer's instructions and sequenced in both directions using an automated sequencer, ABI3130 Sequencer (Applied Biosystems, USA) at the Southern Pathology Sequencing Facility, Flinders Medical Centre (South Australia). 16S rRNA sequences were analyzed using the BLAST programme against the NCBI databases. The highest hit obtained through blastn match for the strain MK2 was used for ClustalW multiple alignment and generating a phylogenetic relationship. The neighbor joining tree was constructed using the molecular evolutionary genetic analysis package version 5.0 based on 1000 bootstrap values (Tamura et al., 2011). The 16S rRNA sequence of strain MK2 was deposited in GenBank under accession number JQ316533.

### Assessment of biodegradation potential and electrochemical activity

The hydrocarbonoclastic potential of strain MK2 was evaluated by measuring the reduction of metabolic indicators such as dichlorophenol indophenol and tetrazolium salts (Pirôllo et al., 2008). Experiments were also conducted to pre-screen the possible candidate electroactive bacterial strains by *in vivo* biodecolourization assay using diazo dyes as stated earlier (Hou et al., 2009). Experiments were carried out in both aerobic and anaerobic environments using 20 ml of nutrient broth with different concentrations (50, 100, 150 mg l^−1^) of an azo dye, Reactive Black5 (RB5). The dye degradation was monitored by observing the decrease in absorbance of suspension at 595 nm under a UV-visible spectroscopy system (Agilent model 8458). All decolorization studies were conducted in triplicate for each experiment, and the activity was expressed as percentage degradation as follows:

$$Percentage\;of\;dye\;decolourization\;=\frac{A_{i}-A_{t}}{A_{i}}\times 100$$

where *A*_*i*_ = initial absorbance and *A*_*t*_ = observed absorbance at designated intervals.

### Hydrocarbon biodegradation experiments

To obtain 1 OD culture, overnight grown bacterial cells were centrifuged for 20 min at 4500 rpm. The cell pellet was washed three times and re-suspended in MSM until the OD_600_ was equivalent to 1.00. One percent of the 1 OD culture of strain MK2 was transferred to 100 ml of MSM with a concentration of 8000 mg l^−1^ of DRO and incubated at 25 °C for time course experiments with shaking at 150 rpm. The cell growth was determined by the comparison of optical density against the control at designated time intervals. Hydrocarbonoclastic potential was also monitored under anaerobic nitrate reducing environments. The inoculum size was 1% of the anaerobically grown bacterial cells with nitrate (10 mM) and 8000 mg l^−1^ of DRO as an electron acceptor and donor, respectively from an anoxic sterile stock solution. All cell cultures were maintained in triplicate for each experiment. All procedures for anoxic growth experiments, from medium preparation to manipulating the strain were performed using standard anoxic conditions. All culturing was done in sealed serum vials with nitrogen/carbon dioxide (80:20, v/v) in the headspace. The sealed vials were incubated at 25°C for time course experiments with shaking at 150 rpm. An uninoculated control was prepared for each set of biodegradation experiments. The samples from the time course experiments of aerobic and anaerobic incubations were extracted three times with 1:1 solvent mixture of acetone-methylene chloride, dewatered and concentrated by an evaporator. The evaporated hydrocarbons were taken as residual hydrocarbons and dissolved in n-hexane, filtered through 0.25 μm membrane filters and analyzed by gas chromatography.

### Fuel cell experiments

#### MFC construction and operational conditions

Single chamber MFC systems were constructed from laboratory bottles (320 ml capacity, Schott) as previously described (Logan et al., 2007) with a modification to increase electrode area. The anode electrodes composed of carbon fiber brushes with wire titanium cores that had an initial surface area of 6.99 m^2^ g^−1^. These fiber electrodes were cleaned by soaking overnight in acetone followed by pre-treatment with sulfuric acid (concentrated, 100 ml l^−1^) and heat treatment to improve the geometric surface area of the electrodes as described by Feng et al. (2010). The cathode was fabricated using flexible carbon cloth coated with a hydrophobic PTFE layer (Cheng et al., 2006) with additional diffusional layers on the air breathing side to cut down fouling rate and evaporation of hydrocarbons. In contrast, the hydrophilic side was coated using a mixture of nafion perfluorinated ion exchange ionomer binder solution, carbon, and platinum catalyst (0.5 g of 10% loading). The electrodes were connected using copper wire with all exposed metal surfaces sealed with a non-conductive epoxy resin (Jay Car, Australia). All the reactors were sterilized before use. Strain MK2 was used for microbial electrochemical experiments with acetate (1 g l^−1^) as the electron donor in 50 mM PBS buffer. The anodic chamber was flushed for 30 min with nitrogen gas before the operation. The anolyte was agitated using a magnetic stirrer operating at 100 rpm. Open circuit MFC studies were also carried out and then switched to the closed circuit with a selected external load (R-1000 Ω unless stated otherwise). Reactive Black 5 was used as sole source of energy in dye degradation experiments using the strain MK2 at a concentration of 50 mg l^−1^ in MFC studies. LB medium was used in biodecolorization studies with an external load of 1000 Ω. MFCs were operated in a fed-batch mode until the voltage fell to a low level (≤10 mV), and then the anolyte solution was replaced under anaerobic chamber (10% hydrogen, 10% carbon dioxide and 80% nitrogen) (Don Whitley Scientific, MG500, Australia) conditions. All the reactors were maintained at room temperature in triplicates.

### Cloning and phylogenetic analysis of possible catabolic genes for hydrocarbon degradation

Genes encoding alkane hydroxylase enzyme complex including *alk* and *rub* genes were amplified by a polymerase chain reaction (PCR) method using oligonucleotides listed in Table S1. The PCR mix of 50 μl contained the following: 10 μl of Gotaq 5X buffer, 2.0 μl of MgCl_2_ (25 mM), 1 μl of dNTP mix (10 mM), 2 μl of each primer (100 mM), 10–15 ng of purified DNA and 2.5 U taq DNA polymerase (Promega, Australia). Cycling was performed with an initial denaturation for 5 min, followed by 35 cycles of 60 s at 94°C, 30 s of annealing at 40–60°C, 60 s of extension at 72°C and a final extension at 72°C for 10 min, using a Bio-Rad thermal cycler. The primers were designed based on the available draft genomes of *S. maltophilia* using Primer—BLAST tool from NCBI and assessed by Oligo 6 software. The amplification products were purified using the UltraClean PCR clean-up kit (Mo Bio, CA) and ligated into the _*P*_GEM-T-Easy vector. After transformation into *E. coli* DH5α individual plasmid inserts were sequenced. *In silico* analysis was done by using the blast programs to search the GenBank and NCBI databases (http://www.ncbi.nlm.nih.gov).

### Analytical methods and calculations

Cell voltage was monitored using a DMM (Keithly Model 2701, USA) linked to a multi-channel scanner (Module 7700, Keithly Instruments, USA). Data were recorded digitally on an Intel computer via IEEE 488 input system and Keithly cable. To measure the current under closed circuit conditions, the external resistance was connected (R-1000 Ω unless stated otherwise). Polarization curves were obtained using various external loads ranging from 10 Ω to open circuit. Current was calculated by using I = V/R. The power density was calculated as follows; where V was the cell voltage, I was electrical current and A denoted the electrode surface area. Power density and current density were normalized to the projected surface area of a cathode (Logan, 2008).

(1) $$P=\frac{\text{V}\cdot \text{I}}{\text{A}}$$

Coulombic efficiency (*CE*) was calculated at the end of the cycle from *COD* removal as follows (Logan, 2008),

(2) $$CE(\%)=\frac{M\int_{0}^{t}I\cdot dt}{Fbq\Delta COD}\times 100$$

where, *M* is the molecular weight of the substrate, *F* = Faraday's constant, *b* = number of electron exchanged/1 M of oxygen, Vn = volume of liquid in the anode chamber, Δ*COD* = difference in the *COD* of initial and end batch samples from MFCs. Graphite fiber surface area was also measured using a Brunauer–Emmett–Teller (BET) isotherm (Mi micromeritics, Gemini V, Particle and Surface Science Pty Ltd.) DRO degradation experiments were conducted using data from triplicate analyses. The DRO was extracted in acetone-methylene chloride (1:1) mixture, dewatered and concentrated by an evaporator, and then analyzed with GC-FID (Flame Ionization Detector) using an HP-5 capillary column (15 m length, 0.32 mm thickness, 0.1 μm internal diameter) (USEPA, 1996). The estimated recovery was more than 70%.The GC programme was set up according to USEPA (USEPA, 1996). The carrier gas was helium. The operational temperature ranged from 50 to 300°C with a programmed temperature gradient of 25°C/min. The resulting chromatograms were analyzed using Agilent software (GC-FID Agilent model 6890) to identify the petroleum degradation products (Venkidusamy et al., 2016).

## Results

### Strain isolation and physiology

**Figure 1:**
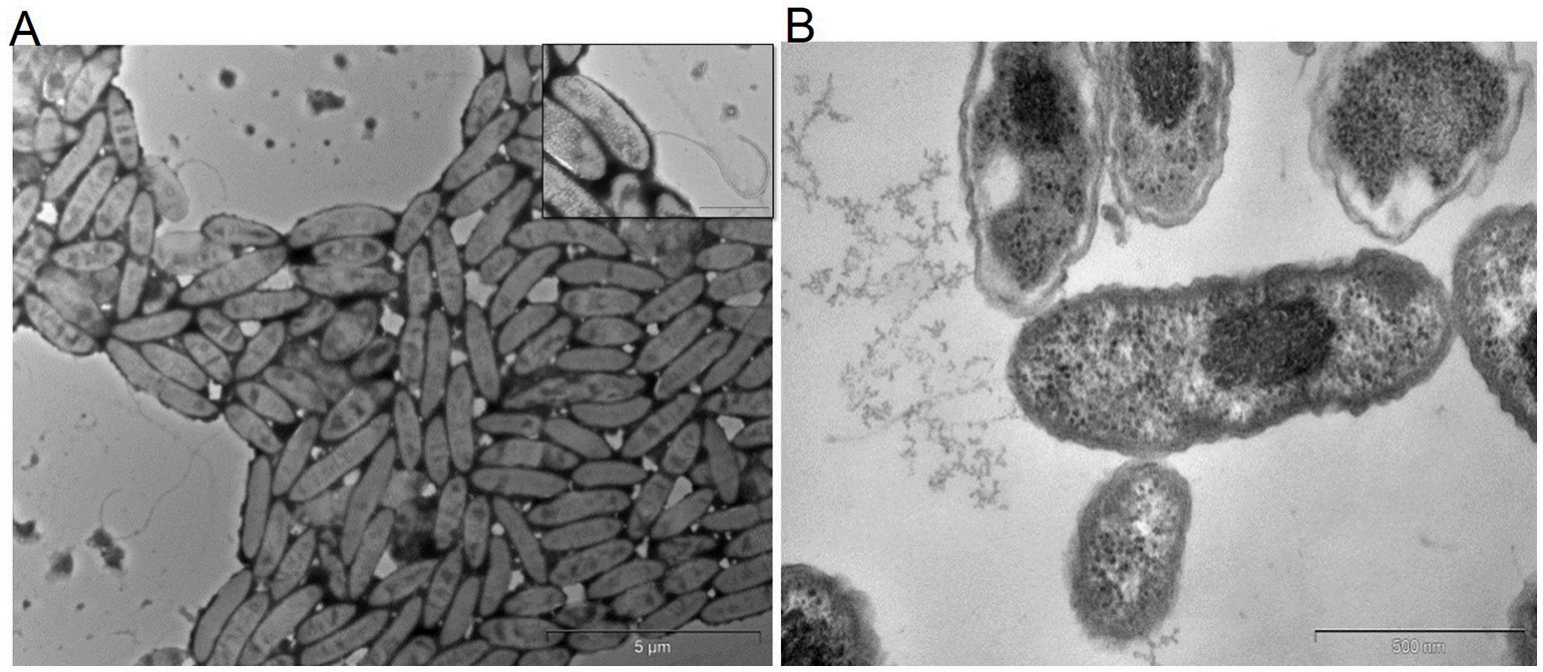
**Transmission electron micrographs of *S. maltophilia* MK2**. Bar scale, 500 nm. **(A)** Cells with flagella. **(B)** Bacillus shaped cells of strain MK2.

From the anodes of enriched PH fed MERS, a pure culture of facultative, hydrocarbonoclastic bacterial strain MK2 was isolated by serial dilution and plating techniques. Cells of strain MK2 contains double membrane bilayers, produces polar flagella in tufts or as single (Figure 1A) and grow as bacillus shaped (Figure 1B). Cell growth on nutrient agar medium produces large gleaming colonies which are pale yellow in color. The bacterial strain grew at temperatures ranging from 25 to 37°C at a neutral pH (optimum temperature 30°C), while no growth was detected above 40°C. The strain was negative for oxidase and catalase is present. The bacterial strain was shown to be capable of anaerobic growth through amino acid fermentation and anaerobic nitrate reduction through quantitative biochemical analysis. However, it was unable to metabolize sugars such as glucose and lactose through the anaerobic fermentation process. Cell growth was accompanied by the strong ammonia odor with pale green discoloration in old LB plates. The strain MK2 displayed a limited nutritional spectrum as highlighted by its genus name (Table S2). The strain was unable to utilize arabinose, adonitol, fructose, xylose, rhamnose, gluconate, etc., Salient properties of the strain MK2 were direct electrode respiration and the ability to degrade n-alkane components of PH in both aerobic and anaerobic environments.

### Phylogenetic analysis and taxonomy

**Figure 2:**
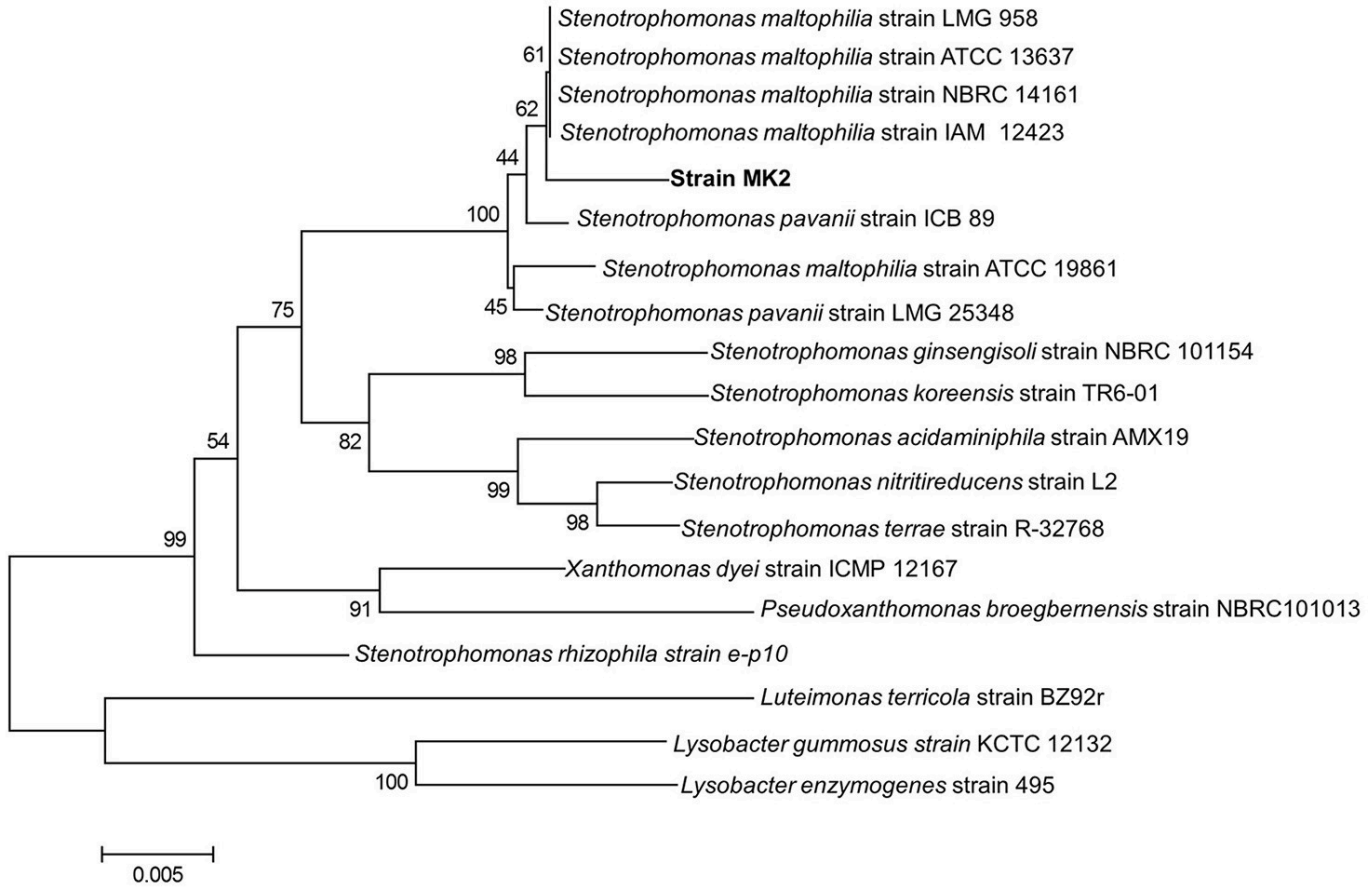
**Phylogenetic tree based on 16S rRNA sequences showing the positions of the strain MK2 and representatives of other *Stenotrophomonas* spp**. The tree was constructed from 1448 aligned bases. Scale bar represents 0.005 substitution per nucleotide position.

An almost complete 16S rRNA gene sequence (1448 bp) was obtained for strain MK2 and analyzed phylogenetically using ClustalW alignment. Using this multiple alignment, the neighborhood phylogenetic tree was constructed (Figure 2). The taxonomic position shows that the strain MK2 was a member of the *Stenotrophomonas* subgroup in the class of γ-proteobacteria. From a BLAST analysis, the highest level of sequence similarity (98%) matched with *Stenotrophomonas maltophilia strain* ATCC 13637.

### Redox indicator assays for the assessment of hydrocarbonoclastic potential

The hydrocarbonoclastic potential of strain MK2 was assessed through a preliminary investigation of hydrocarbon consumption, a concomitant increase in biomass and reduction of redox electron acceptors such as DCPIP and tetrazolium indicators. The strain MK2 discolored the redox indicator from the blue to violet during the first 24 h and complete discoloration was observed by the end of 120 h when DRO was the sole carbon and energy source. Also, the formation of a red precipitate formazan from the tetrazolium was observed while the abiotic controls remained unchanged. It is evident from the above screening assays that the strain MK2 can utilize diesel derived hydrocarbons.

### Screening assays for the assessment of electrochemical activity

**Figure 3:**
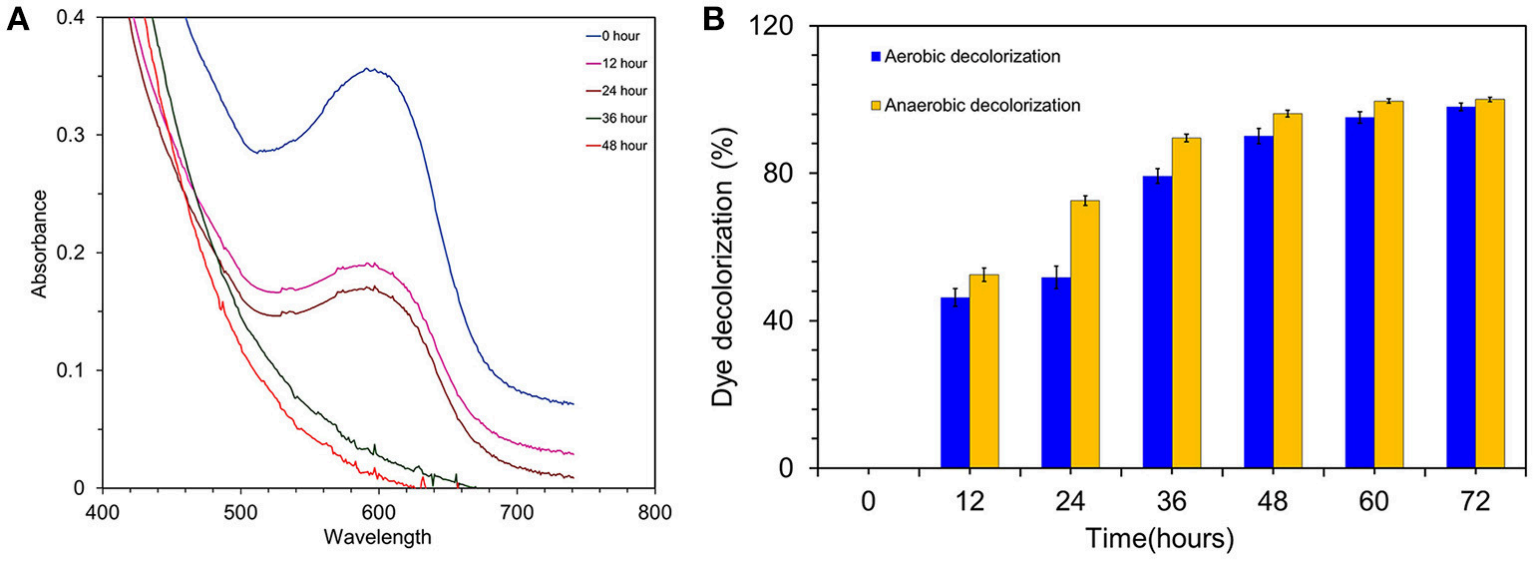
**(A)** Time overlaid absorbance spectra of RB5 biodecolourization by the strain MK2. **(B)** Biodecolourization of diazoic dye RB5 by the strain *S. maltophilia* MK2 under aerobic and anaerobic environments.

To pre-screen the electrochemical activity of the strain MK2, aerobic and anaerobic cultures were grown in nutrient broth supplemented with 50 mg l^−1^ of RB5. This concentration was found to be supportive for a higher growth rate and rapid decolorization among the various concentration of RB5 tested. The complete disappearance of the characteristic absorption peak at the region of λmax (597 nm) and simultaneous decolorization were observed in aerobic and anaerobically incubated samples (Figure 3). Figure 3A shows dynamic changes of the absorption spectra observed during the decolorization process under anaerobic conditions. RB5 azoic dye was almost completely decolorized (96.23%) in 48 h by *S. maltophilia* MK2 under anaerobic environments while it took 72 h for nearly complete decolorization (97.99%) under aerobic conditions (Figure 3B). The blue pigmented dead cell pellet from the heat-killed cells in the control showed a passive adsorption of dye, whereas colorless cell pellets obtained from the living cultures demonstrated that reduction of the RB5 indicator had occurred.

### Energy generation by *S. maltophilia* MK2 in microbial electrochemical cells

#### Current generation in acetate fed MFCs

**Figure 4:**
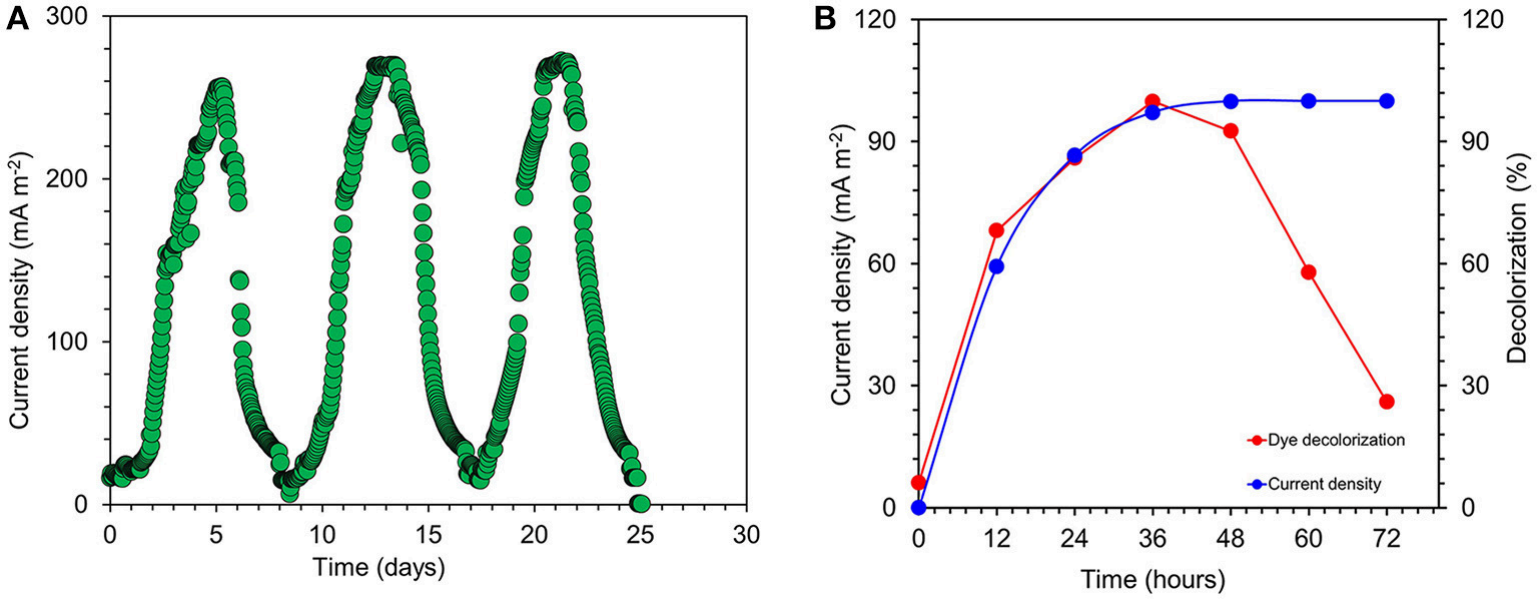
**(A)** Few representative cycles of current density generated by *S. maltophila* MK2 in acetate fed microbial fuel cells. **(B)** Current generation and simultaneous dye decolorization in dye fed MERS using *S. maltophila* MK2.

Current was generated in all the MFCs inoculated with *S. maltophilia* MK2 within a few hours using acetate as an energy source. After 3 days, voltage started to follow a constant pattern and then stabilized. The fuel cell electrodes were connected through a resistor (*R* = 1000 Ω) once it reached the plateau voltage generation stage. The maximum output range of voltage and current density were 414 ± 7 mV, 273 ± 8 mA/m^2^ (*R* = 1000 Ω) after four cycles of operation. After five refilling batches with a fresh substrate, the maximum current output of each batch became stable (270 ± 5 mA/m^2^). Few representative cycles (average current density from triplicates) of current density are shown in Figure 4A. The maximum CE was 34.8% which corresponded to the maximum current density of 272.96 mA/m^2^.

#### Current generation and simultaneous dye decolorization in dye fed microbial electrochemical cells

The current was rapidly generated in azo dye fed MFCs inoculated with *S. maltophilia* MK2 cells within few hours of using azoic dye as an energy source at 1000 Ω. The maximum output range of voltage and current density were 145 ± 6 mV, 94 ± 6 mA/m^2^. Constant and repeatable power cycles were obtained during five changes of the contents of the anode chamber. Using RB5 concentration of 100 mg l^−1^ in MFC, 59.3 ± 1.25% was removed during the first 12 h of operation. After 24 h, almost 97.2 ± 1.64% of RB5 was decolorized and it was below detection limits at the end of the batch operation when the voltage of the batch reached >10 mV as shown in Figure 4B.

### Hydrocarbonoclastic potential of *S. maltophilia* MK2

#### Aerobic biodegradation of DRO

**Figure 5:**
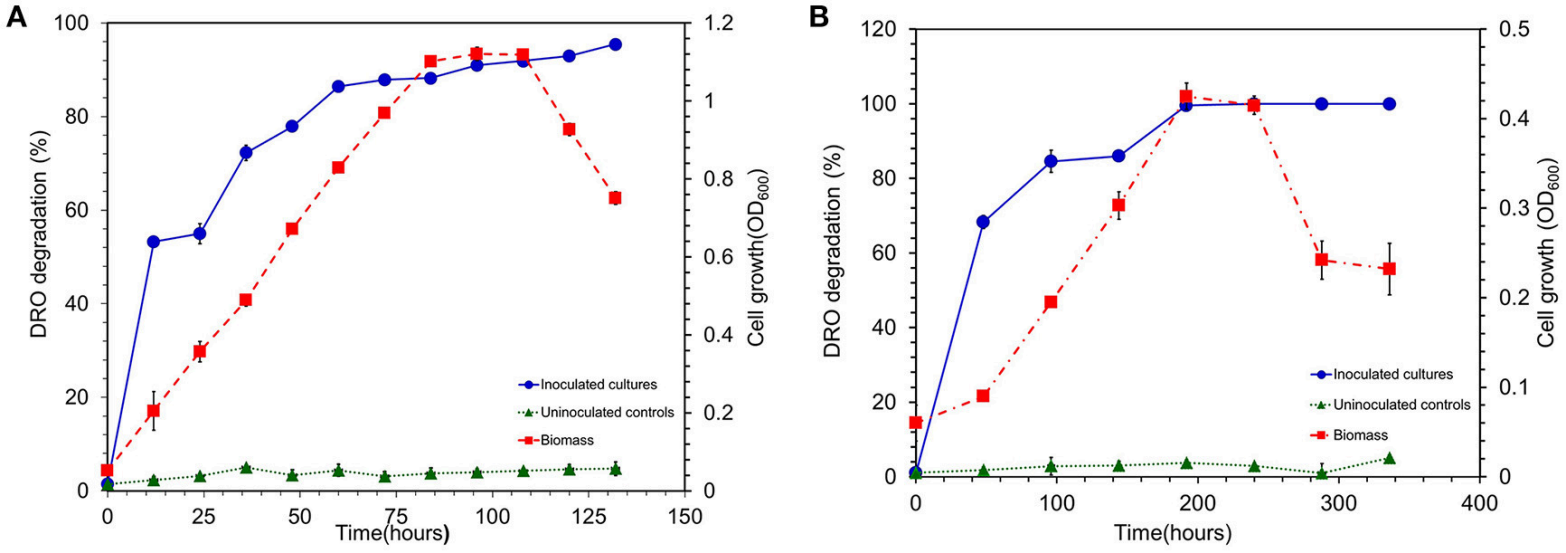
**(A)** Biodegradation of DRO compounds by aerobically grown cells of *S. maltophila* MK2 (Blue circle shows DRO degradation in MK2 inoculated samples; Red square shows the biomass density; Green triangle shows the DRO degradation in uninoculated controls). **(B)** Biodegradation of DRO compounds by anaerobically grown cells of *S. maltophila* MK2 (Blue circle shows DRO degradation in MK2 inoculated samples; Red square shows the biomass density; Green triangle shows the DRO degradation in uninoculated controls).

To evaluate the hydrocarbon degradation potential of the strain MK2, experiments were performed under two different environments *viz.*, aerobic and anaerobic. The rate and extent of biodegradation were interpreted from GC chromatograms of the residual hydrocarbons. The aerobic incubation experiments indicated that the biodegradation of hydrocarbons by strain MK2 was more efficient than anaerobic incubations. Figure 5A shows the possible cell growth and its associated substrate degradation by strain MK2. For a substrate concentration of 8000 mg l^−1^, cells started growing within 24 h with a rapid decrease in DRO concentration of about 53%. After 84 h, the strain reached as second peak of growth while the DRO degradation was 88%. The temporal removal of DRO reached >90% after 100 h of incubation. In general, the rate of degradation increased consistently with increasing cell biomass during the early stage of the exponential phase and then, it reached a plateau at stationary and death phase of cell growth. Abiotic loss of DRO was measured under each stage was less than 5%. The GC profile of the residual n-alkanes of DRO after the incubation was compared with that of the original as shown in Figure S1. At the end of the incubation period (150 h), the n-alkane members of C8 to C36 were almost completely metabolized in the samples inoculated with the strain MK2.

### Anaerobic biodegradation of DRO

The hydrocarbonoclastic activity of the strain MK2 was examined under anaerobic conditions with DRO as the sole source of carbon and nitrate as the final electron acceptor. The results indicated that the biodegradation of DRO (8000 mg l^−1^) in anaerobic environments is slower in comparison to the aerobic degradation. Figure 5B shows the quantitative growth experiments with depletion of DRO at a time course within 14 days. The growth of strain MK2 was slow until 96 h of incubation and then reached a log phase by 100 h. The hydrocarbonoclastic potential was closely coincided with the phase of cell growth, as a result, degradation efficiency increased from the 2nd day to the 8th day of incubation, before leveling off from the 10th to the 12th day. By the 10th day, a complete degradation of the substrate had occurred. Figure S2 depicts the residual DRO concentration before and after incubation under anaerobic conditions.

#### Detection of possible catabolic genes involved in hydrocarbon degradation

**Figure 6:**
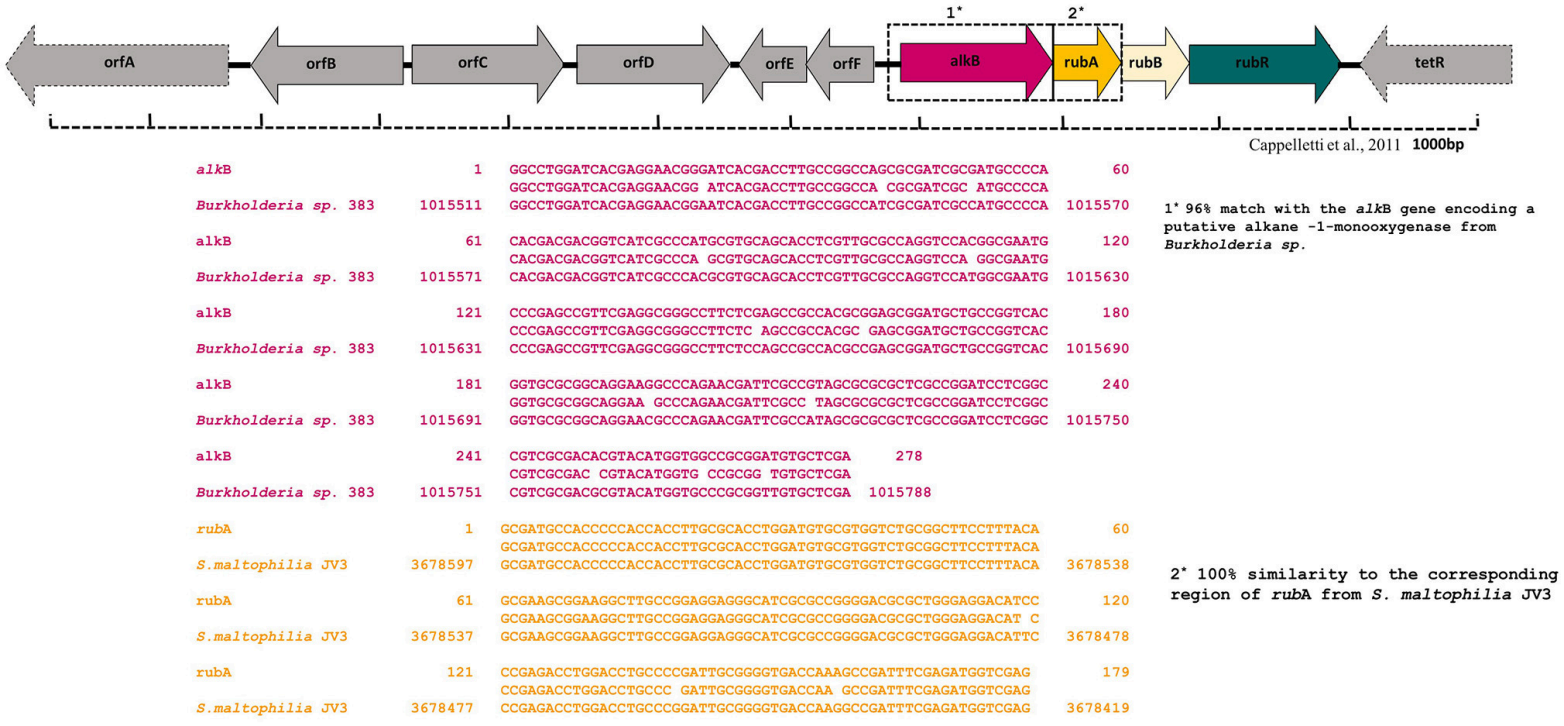
**Alignment of alkane monooxygenase (*alk*B) and rubredoxin (*rub*A) gene sequences generated using CLUSTALW multiple alignment in *S. maltophilia* MK2**.

The presence of specific catabolic genes (*alk*B and the related, *alk*M, *alk*A) encoding alkane hydroxylase enzyme complex was investigated by a PCR-mediated amplification with various oligonucleotide primers. Of the 15 different oligonucleotides combinations tested for PCR amplification, only the primer combination of the ALK3 set provided a positive result. Blastn searches in the GenBank database showed that the PCR product was similar to a number of known *alk*B genes, had a 96% match with the *alk*B gene encoding a putative alkane -1-monooxygenase from *Burkholderia* (Figure 6). In order to explore the presence of other functional genes from the strain MK2, new primers were designed to amplify the second cluster of the alkane hydroxylase complex (Table S1). A PCR product of the expected size was obtained when the primer combinations *rub*F, *rub*R used. Blastx alignments showed that this PCR product had 100% similarity to the corresponding region of the *S. maltophilia* rubredoxin type Fe(Cys)4 protein (Figure 6).

## Discussion

The enrichment of hydrocarbonoclastic Electrode respiring bacteria (ERB) able to utilize hydrocarbons as a sole source of carbon and energy in MERS led to the isolation of hydrocarbonoclastic bacterial strain identified as *S. maltophilia* MK2. *Stenotrophomonas* spp. are often considered to be ubiquitous, however, these species are frequently found in marine, soil, rhizosphere of diverse plants (Denton and Kerr, 1998) and polluted environments (Binks et al., 1995; Dungan et al., 2003; Lü et al., 2009) as their main environmental reservoirs. The representative candidate, *S. maltophilia* MK2 is a free living, facultatively anaerobic bacterium and phylogenetically placed in the phylum of Proteobacteria, Gammaproteobacteria, Xanthomonadales, Xanthomonodaceae (Palleroni and Bradbury, 1993). The environmental isolate *S. maltophilia* MK2 reduces nitrate in anoxic environments as reported earlier in some strains of this genus (Woodard et al., 1990). However, the additional distinctive features make this strain different from the existing members of the family include (i) growth by anaerobic fermentation of the amino acids present in tryptone and peptone (ii) electrochemically active under acetotrophic environments (iii) ability to degrade n-alkane components of DRO in anaerobic conditions (iv) Biodecolorization of synthetic dyes. The regular growth mode of this bacterial strain *S. malotophilia* MK2 is aerobic heterotrophy; however, the strain MK2 can grow in anaerobic environments either through amino acid fermentation or nitrate reduction. The previous studies on strain ZZ15 belongs to *S. maltophilia* showed a microaerophilic growth under denitrifying environments (Yu et al., 2009). In contrast, the pure cultures of many *Stenotrophomonas* strains are unable to grow in oxygen lacking conditions (Assih et al., 2002; Dungan et al., 2003).

### Metabolic versatility vs. environmental bioremediation

#### Bioremediation potential

The genus *Stenotrophomonas* has been studied as a promising candidate for biotechnological applications involved in the detoxification of various man-made pollutants because of its broad spectrum of metabolic properties (Ryan et al., 2009). These include utilization of N-aromatic rings (Boonchan et al., 1998), alkyl benzene sulfonates of organophosphate pesticides (Dubey and Fulekar, 2012), phenyl urea herbicides (Lü et al., 2009), chlorinated compounds (Somaraja et al., 2013), heavy metals (Pages et al., 2008; Ghosh and Saha, 2013) and other groups of xenobiotic pollutants (Tachibana et al., 2003; Li et al., 2012). Aliphatic hydrocarbons including straight and cycloalkanes, unsaturated hydrocarbons and aromatic hydrocarbons, are the building blocks of diesel oil (Air Force, 1989) and n-alkanes are the most dominant fraction. The degradation of these hydrocarbon compounds in anoxic environments by the genus *Stenotrophomonas* is previously unknown. Here, we demonstrate for the first time evidence for the occurrence of hydrocarbonoclastic behavior in the strain MK2 under anaerobic, nitrate reducing environments.

The preliminary screening assays reveal that the strain MK2 possess the hydrocarbonoclastic potential by involving redox reactions in which electrons are donated to terminal electron acceptors during the cell respiration. The reduction of a lipophilic mediator such as DCPIP (blue to colorless) coupled with the formation of oxidized products showed that the biodegradation had been carried out by metabolically active cell growth, not by adsorption to cells associated with the water-carbon interface (Kubota et al., 2008). The respiratory reduction of tetrazolium salts is another criterion employed by many researchers (Olga et al., 2008; Pirôllo et al., 2008) to determine the dehydrogenase activity of hydrocarbonoclastic bacterial strains. Upon reduction of this salt, the color changed to red due to the formation of insoluble formazans by the production of superoxide radicals and electron transport in the bacterial respiratory chain (Haines et al., 1996). In order to corroborate the potential hydrocarbon degradation by the strain MK2, GC scan was performed using heterotrophically incubated samples grown under aerobic and anaerobic conditions. The highest rate of degradation of the light end hydrocarbons of DRO was observed at 24 h with aerobic incubations, whereas this tended to be slower (96 h) under anaerobic conditions. GC resolved n-alkanes from C8 to C36 peaks (Figure S1) in inoculated samples demonstrated the occurrence of the enhanced hydrocarbon degradation when the bacterial strain grown under the aerobic conditions. It was quite possible to achieve a complete degradation of DRO under aerobic conditions by appropriately increasing the incubation time of the experiment. Such hydrocarbonoclastic behavior is in contrast to the earlier findings of Saadoun (2002) and Ueno et al. (2007) where their strain of *S. maltophilia* was unable to degrade hydrocarbons as a sole carbon source. On the other hand, members of this genus have been found along with other predominant genera of hydrocarbon degraders including *Acinetobacter, Pseudomonas, Alcaligenes, Sphingomonas* in oil contaminated environments as stated earlier (Van Hamme et al., 2000; Zanaroli et al., 2010). The previous studies on the microbial electrochemical remedial process of hydrocarbons have also demonstrated the ubiquity of *Stenotrophomonas* spp. and their dominance in the anodic microbial communities (Morris et al., 2009; Venkidusamy et al., 2016). The capability of hydrocarbon degradation has also been demonstrated earlier in a soil isolate of *S. maltophilia* strain DJLB only under aerobic conditions (Ganesh and Lin, 2009). It is of interest that, the present study reveals the complete mineralization of n-alkane members of DRO (C8–C36) for the first time, including the biomarkers pristine, phytane, and a short chain to long chain aliphatic hydrocarbons under anaerobic incubations by the strain MK2 during a 12 days period in the presence of nitrate.

### Exoelectrogenic potential

The characteristics of diazoic dye decolorization were used as a simple criterion for pre-screening the possible electrochemically active microbial candidates in the present study as stated earlier (Hou et al., 2009). The present study showed the simultaneous decolorization and decreased dye concentrations from batch culture studies of anaerobic and aerobic incubations with strain MK2 inoculum. The efficiency of color removal was more than 95% under anaerobic conditions as reported in another exoelectrogenic strain of *Shewanella* spp. (Pearce et al., 2006). This is in agreement with the previous studies on the assessment of electrochemically active microbial strains using MFC arrays (Hou et al., 2009). Dye decolorization occurs because of a reductive electrophilic cleavage of the chromophore, a functional group of azo linkage, by biocatalysts as reported earlier (Sun et al., 2009; Satapanajaru et al., 2011). To confirm the extracellular access to the insoluble electron acceptors, the exoelectrogenic property of the strain MK2 was also investigated in two different environments (i) acetotrophic (ii) dye decolorization, using microbial electrochemical systems. The present study exhibited a maximum power density of 113 ± 7 mW/m^2^ with a recovery of 34.8% as an electrical current using the strain MK2 in acetotrophic conditions. In the case of the reactors fed with azoic dye demonstrated the potential of generating current (99.93 ± 6 mW/m^2^) with the concurrent decolourization using the strain MK2 in MFCs for the first time. The results presented in this study suggest that the strain MK2 is capable of utilizing insoluble electron acceptors such as electrodes through extracellular electron transfer mechanisms. Recent investigations have revealed the potential of using such pure cultures of heterotrophic biofilms in microbial electrochemical remediation cells for dye detoxification (Chen et al., 2010a,b). For instance, Chen et al. (2010a), reported the possibility of using pure cultures of *Proteus hauseri* in MFC, however, decolorization efficiency and power densities generated were much lower. The performance of these microbial electrochemical cells using pure cultures of exoelectrogens are considerably affected by a number of reactor parameters and operating conditions as reported earlier (Min et al., 2005; Logan, 2008).

### Genetic potential

**Figure 7:**
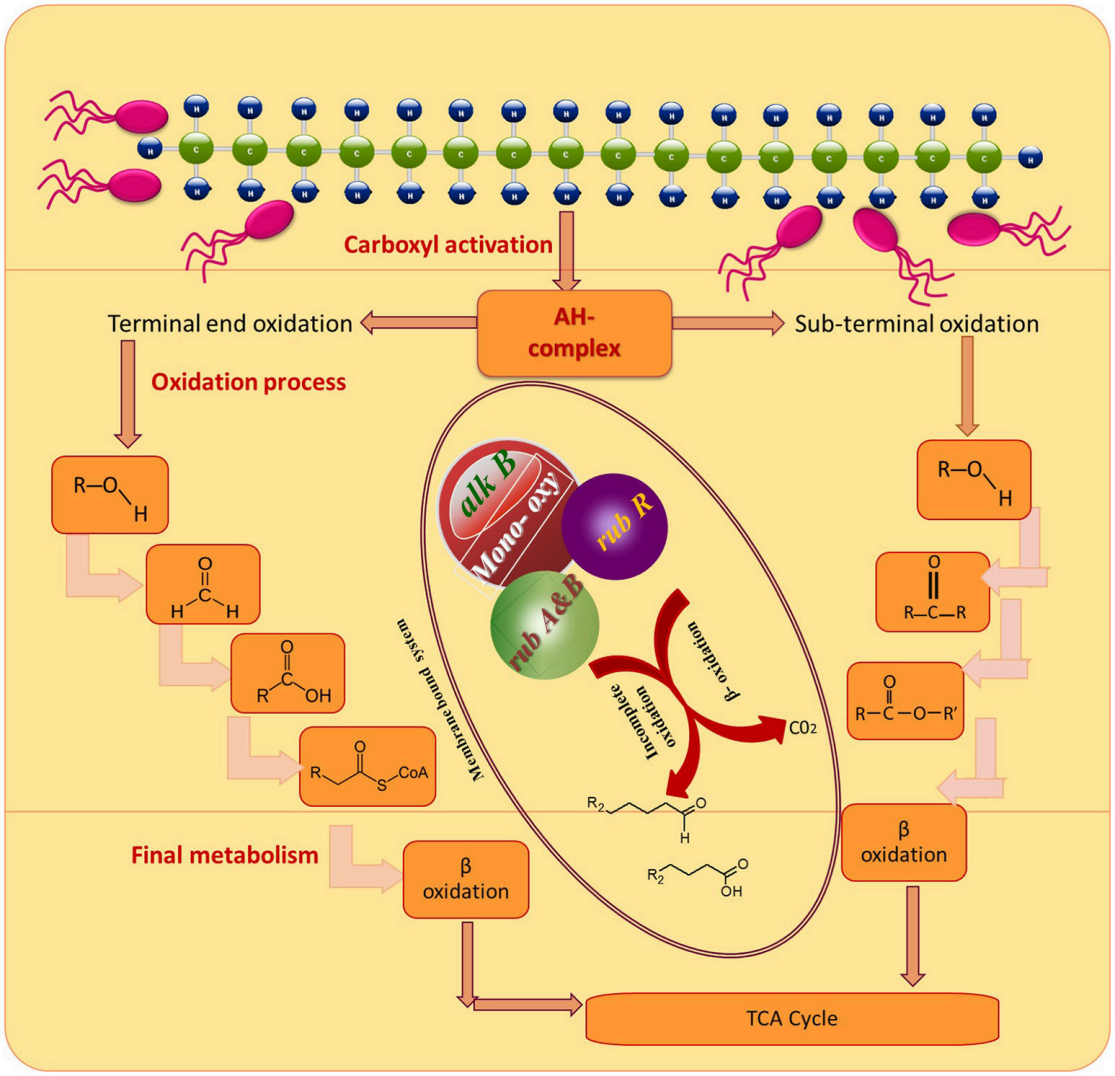
**Degradation pathways of DRO compounds in aerobic environments**.

To gain deeper insights into the hydrocarbon degradation mechanism by the strain MK2, we carried out a gene specific PCR analysis to identify the possible catabolic genes encoding alkane degrading enzymes using different degenerate oligonucleotides. The mechanisms of these alkanes activation vary according to the lifestyle of representative candidate microorganisms and growth environments. Under aerobic environments, the biodegradation typically occurs through a sequential oxidation of n-alkanes resulting in corresponding alcohol and aldehydes groups. These aldehydes further metabolized into fatty acids and conjugated with CoA through β oxidation process which then enter into the tricarboxylic acid cycle as shown in Figure 7 (Van Hamme et al., 2003; van Beilen et al., 2004). Such a successional oxidation process is activated by a family of integral membrane proteins called alkane hydroxylase enzyme system, and this was first studied in *Pseudomonas putida* GPo1. This is of particular interest being a three component biocatalyst and composed of alkane monooxygenase (*alk*B group), dinuclear iron rubredoxins (*rub*A, *rub*B) and mononuclear rubredoxin reductase (*rub*R) (Rojo, 2009; Teimoori et al., 2011). These genes encode the alkane hydroxylase (*alk*) system in the enzyme complex which activates the terminal carbon atoms in the chain of hydrocarbons. While searching the catabolic genes that encode *alk* system in the strain *S. maltophilia* MK2, we found for the first time a conserved chromosomal region of *alk*B and *rub*A (Figure 6). Insilco analysis of this gene showed that the *alk*B region was highly similar to the region observed from an *alk*B gene of *Burkholderia* spp. and this is presumably the gene providing this activity, supporting the close relationship between *S. maltophilia* and *Burkholderia* spp. (84%) at the genomic level. This result suggests that the genes involved do not correspond in terms of their sequence to the same genes as per the published *Stenotrophomonas malotophilia* genome and were instead derived from some other organism with different gene sequences (and the discovery that the *alk*B gene sequence comes from *Burkholderia* supports this). In contrast, the earlier studies on catabolic genes for alkane degradation in *Stenotrophomonas* spp. have shown negative results for the amplification of *alk*B gene (Smits et al., 1999; Vomberg and Klinner, 2000). The presence of a *rub*A gene with 100% homology to Rubredoxin-type Fe(Cys)4 protein of *S. maltophilia* R551-3, shows that the bacterial strain MK2 likely possesses an essential electron transfer components for alkane hydroxylation. Together, these results perhaps indicate the presence of the two conserved domains of *alk*B-*rub*A fused proteins in a contiguous open reading frame as shown earlier in metagenomic analysis of *alk* genes of different microbial genomes from diverse environments (Nie et al., 2014). Such a fusion would be responsible for the extended spectrum of alkane degradation up to C36 hydrocarbons shown in the present study, as *alk*B often reported to be responsible for <C16 length (van Beilen and Funhoff, 2007). Also, the earlier studies on the disruption of the *rub*A encoding genes in bacterial strains lead to the failure of alkane degradation (Haspel et al., 1995; Ratajczak et al., 1998) which supports the aforementioned theory on fused proteins in hydrocarbon biodegradation.

Our present findings demonstrate the facultative hydrocarbonoclastic and exoelectrogenic properties in different environments (acetotrophic and dye detoxification) associated with genus *S. maltophilia* MK2. These species include both environmental non-pathogenic isolates and opportunistic clinical isolates of nosocomial origin. An increasing number of studies demonstrate the potential use of opportunistic human pathogenic bacteria in microbial electrochemical systems that include, *Ochrobactrum anothropi* (Zuo et al., 2008), *Pseudomonas aeruginosa* (Venkataraman et al., 2010), *Acrobacter butzleri* (Fedorovich et al., 2009). The production of lipopolysaccharides, flagella or fimbriae —mediated interactions (Brooke, 2012) may involve in colonization and biofilm formation on the electrodes of microbial electrochemical systems, however their role, lifestyle and pathogenicity of the strain MK2 await further research. It will be interesting to examine the molecular mechanism of such biofilm attachment to electrodes and extracellular electron transfer potential of this strain MK2.

## Conclusions

The members of *Stenotrophomonas* spp. are found to be cosmopolitan opportunists as their presence often been detected in soil and water systems. Our findings demonstrate that the hydrocarbonoclastic and exoelectrogenic potential associated with genus *Stenotrophomonas* is novel and expands the range of microbial phyla known to degrade hydrocarbon contaminants. The strain is notable in that its *alk*B gene seems to have been derived from *Burkholderia* while its *rub*A gene originated from *Stenotrophomonas*. Additionally, the decolorization of diazoic dyes also makes a supplement to the phylogeny knowledge on bioleaching agents and showing further potential in the treatment of wastewater from textile industries using MERS. On a global scale, the strain provides many exciting opportunities for increasing our understanding on bioremediation that underpins the molecular mechanism of contaminant degradation in a relevant environmental context.

## Author contributions

KV and MM proposed the study. KV conducted the experiments under the supervision of MM. KV prepared the draft with contributions from MM.

### Conflict of interest statement

The authors declare that the research was conducted in the absence of any commercial or financial relationships that could be construed as a potential conflict of interest.
