# Supplementary material for: Identification of Electrode Respiring, Hydrocarbonoclastic Bacterial Strain Stenotrophomonas maltophilia MK2 Highlights the Untapped Potential for Environmental Bioremediation
Source: Front Microbiol. 2016 Dec 9;7:1965. doi: 10.3389/fmicb.2016.01965 (PMC5145854; doi:10.3389/fmicb.2016.01965)
Supplement: Supplementary file 1 [file DataSheet1.DOCX]

Supplementary Material

**Identification of electrode respiring, hydrocarbonoclastic bacterial strain *Stenotrophomonas maltophilia* MK2 highlights the untapped potential for environmental bioremediation**

**Krishnaveni Venkidusamy^a,b*,^ Mallavarapu Megharaj^a,b,c^**

**Correspondence:**  Dr. Krishnaveni Venkidusamy, [krishnaveni.venkidusamy@mymail.unisa.edu.au](mailto:krishnaveni.venkidusamy@mymail.unisa.edu.au)

**Supplementary Figures**

**Table S1. Oligonucleotide primers used in this study.**

| Primers | Sequence(5’-3’) | PCR fragment size (bp) | References |
| --- | --- | --- | --- |
| ALK1F  ALK1R  ALK2F  ALK2R  ALK3F  ALK3R  alkBF  alkBR  alkB1F  alkB1R  alkB2F  alkB2R  alcB1F  alcB1R  alkbF  alkbR  alkFGf  alkFGr  alkMup  alkMDn  DEGF  DEGR  S.alkB3F  S.alkB3R  S.alkB1F  S.alkB1R  alkAF  alkAR  rub F  rub R | CATAATAAAGGGCATCACCGT  GATTTCATTCTCGAAACTCCAAAC  GAGACAAATCGTCTAAAACGTAA  TTGTTATTATTCCAACTATGCTC  TCGAGCACATCCGCGGCCACCA  CCGTAGTGCTCGACGTAGTT  GCGCAAGCTTCCGATTAGCTCAG  GCACTCTTTGTGAGAGAATTCAAC  AAYACNGCNCAYGARCTNGGNCAYAA  GCRTGRTGRTCNGARTGNCGYTG  TGGCCGGCTACTCCGATGATCGGAATCTGG  CGCGTGGTGATCCGAGTGCCGCTGAAGGTG  CGGGGTTCAAGGTCGAGCAT  CAGGACCAGGTTGGTGAAGA  CCGGTGTGGTGGTGGATCGG  GCGAGCRTGATCATGCT  CACGCAGAGCTCGGCGGTCG  GGCTAGGCGAATTCGTATGGTC  CGGGGTAAGCATGAATAGCT  CGTACAGCTACTTGGTGGAC  CGACCTGATCATGCCATGACCGA  TCTAGGTCAGTACACGGTCA  GTGCACTTGTTGGTGGGGGA  CCGCATAACCCCAAGCACGA  ACCCCAGATTGGCGTTCTCC  TGCGATCCAACGCTGATGCC  TGGGTATGGGACTGGCAAGCGA  AGTGAACGGCGCGCAGACAG  GCTGCACCATCTGGAAATCGGCTT  ATGAGCGATGCCACCCCCAC | 185  271  330  204  550  870  434  600  500  496  238  200  400  192  194 | (Kloos et al., 2006)  (Kloos et al., 2006)  (Kloos et al., 2006)  (Wang et al., 2010)  (Kohno et al., 2002)  (Kohno et al., 2002)  (Kohno et al., 2002)  -  -  (Smits et al., 1999)  (Wang et al., 2010)  This study  This study  This study  This study |

**Table S2. Phenotype and metabolic properties of strain MK2 and *S.maltophilia* strain ZZ15**

(+): Positive reaction; (-): Negative reaction; (±): Reaction is not stable; (NR): Not reported

| **PARTICULARS** | ***Stenotrophomonas maltophilia* MK2** | ***Stenotrophomonas maltophilia* strain ZZ15 (Yu et al., 2009)** |
| --- | --- | --- |
| Cell diameter(µm) | 0.5-1 µm | 0.5-2.0 µm |
| Cell shape | Rod | Rod |
| Motility | + |  |
| Optimum pH | 6.5-7.4 | 6.5-7.0 |
| Indophenol oxidase | - | NR |
| Catalase | + |  |
| Glucose fermentation | - |  |
| Lactose fermentation | - |  |
| Tween hydrolysis | + | + |
| Urea hydrolysis | + |  |
| Nitrate-reduction | + |  |
| Denitrification | - | - |
|  | + | + |
| DRO degradation | + | NR |
| Acetate |  |  |
| Arabinose | - | ± |
| Adonitol | - | ± |
| Cellobiose | + | ± |
| Fructose | - | ± |
| Maltose | + | ± |
| Mannitol | - | + |
| Xylose | - | - |
| Citrate | + | - |
| Rhamnose | - | + |
| Gluconate | - | + |
| N-Acetylglucosamine | + | + |
| Sulfide production | - |  |

**Figure. S1. GC finger printing of the residual n-alkanes of DRO compounds (A) before incubation (0hour) (B) after 120 hours) incubation under aerobic conditions**


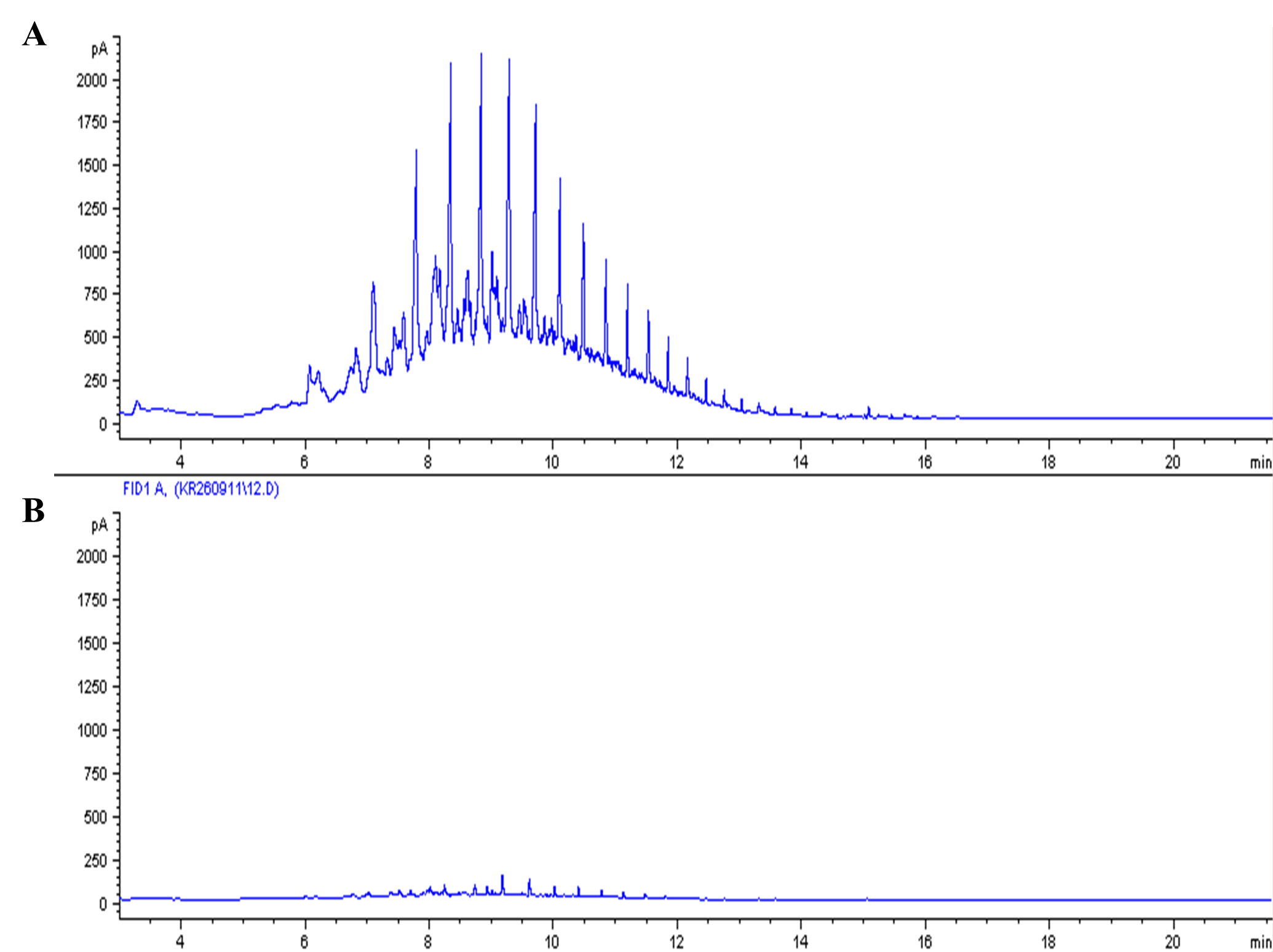


**Figure. S2. GC finger printing of the residual n-alkanes of DRO compounds (A) before incubation (0 hour) (B) after 336 hours incubation under anaerobic conditions.**


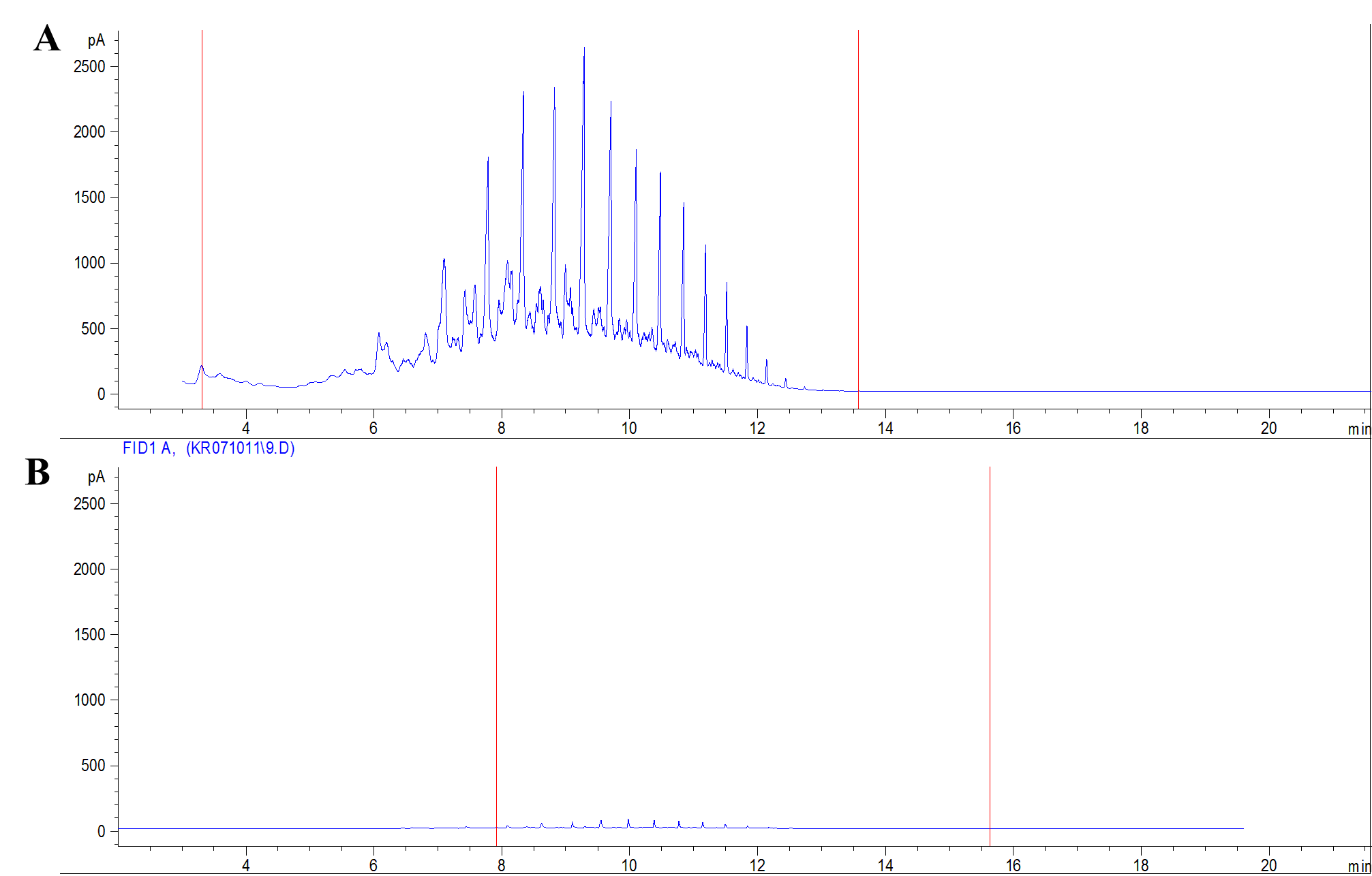


**References:**

Kloos, K., Munch, J.C., and Schloter, M. (2006). A new method for the detection of alkane-monooxygenase homologous genes (*alk*B) in soils based on PCR-hybridization. *J. Microbiol. Methods* 66**,** 486-496.

Kohno, T., Sugimoto, Y., Sei, K., and Mori, K. (2002). Design of PCR Primers and Gene Probes for General Detection of Alkane-Degrading Bacteria. *Microbes. Environ.*17**,** 114-121.

Smits, T.H., Röthlisberger, M., Witholt, B., and Van Beilen, J.B. (1999). Molecular screening for alkane hydroxylase genes in Gram‐negative and Gram‐positive strains. *Environ. Microbiol.*1**,** 307-317.

Wang, W., Wang, L., and Shao, Z. (2010). Diversity and abundance of oil-degrading bacteria and alkane hydroxylase (*alk*B) genes in the subtropical seawater of Xiamen Island. *Microbial. Ecol.*  60**,** 429-439.

Yu, L., Liu, Y., and Wang, G. (2009). Identification of novel denitrifying bacteria *Stenotrophomonas* sp. ZZ15 and *Oceanimonas* sp. YC13 and application for removal of nitrate from industrial wastewater. *Biodegradation.* 20**,** 391-400.
